# A proposed panel of biomarkers of healthy ageing

**DOI:** 10.1186/s12916-015-0470-9

**Published:** 2015-09-15

**Authors:** Jose Lara, Rachel Cooper, Jack Nissan, Annie T Ginty, Kay-Tee Khaw, Ian J Deary, Janet M Lord, Diana Kuh, John C Mathers

**Affiliations:** Human Nutrition Research Centre, Institute of Cellular Medicine and Newcastle University Institute for Ageing, Newcastle University, Biomedical Research Building, Campus for Ageing and Vitality, Newcastle upon Tyne, NE4 5PL UK; MRC Unit for Lifelong Health and Ageing, MRC Unit for Lifelong Health and Ageing at UCL, 33 Bedford Place, London, WC1B 5JU UK; Centre for Cognitive Ageing and Cognitive Epidemiology, University of Edinburgh, 7 George Square, Edinburgh, EH8 9JZ UK; School of Sport, Exercise and Rehabilitation Sciences, University of Birmingham, Edgbaston, Birmingham, B15 2TT UK; University of Cambridge, Addenbrooke’s University Hospital, Cambridge, UK; MRC-ARUK Centre for Musculoskeletal Ageing Research, University of Birmingham, Edgbaston, Birmingham, B15 2TT UK

**Keywords:** Biomarkers, Ageing, Physical capability, Cognitive function, Physiological function, Musculoskeletal function, Endocrine function, Immune function

## Abstract

**Background:**

There is no criterion reference for assessing healthy ageing and this creates difficulties when conducting and comparing research on ageing across studies. A cardinal feature of ageing is loss of function which translates into wide-ranging consequences for the individual and for family, carers and society. We undertook comprehensive reviews of the literature searching for biomarkers of ageing on five ageing-related domains including physical capability and cognitive, physiological and musculoskeletal, endocrine and immune functions. Where available, we used existing systematic reviews, meta-analyses and other authoritative reports such as the recently launched NIH Toolbox for assessment of neurological and behavioural function, which includes test batteries for cognitive and motor function (the latter described here as physical capability). We invited international experts to comment on our draft recommendations. In addition, we hosted an experts workshop in Newcastle, UK, on 22–23 October 2012, aiming to help capture the state-of-the-art in this complex area and to provide an opportunity for the wider ageing research community to critique the proposed panel of biomarkers.

**Discussion:**

Here we have identified important biomarkers of healthy ageing classified as subdomains of the main areas proposed. Cardiovascular and lung function, glucose metabolism and musculoskeletal function are key subdomains of physiological function. Strength, locomotion, balance and dexterity are key physical capability subdomains. Memory, processing speed and executive function emerged as key subdomains of cognitive function. Markers of the HPA-axis, sex hormones and growth hormones were important biomarkers of endocrine function. Finally, inflammatory factors were identified as important biomarkers of immune function.

**Summary:**

We present recommendations for a panel of biomarkers that address these major areas of function which decline during ageing. This biomarker panel may have utility in epidemiological studies of human ageing, in health surveys of older people and as outcomes in intervention studies that aim to promote healthy ageing. Further, the inclusion of the same common panel of measures of healthy ageing in diverse study designs and populations may enhance the value of those studies by allowing the harmonisation of surrogate endpoints or outcome measures, thus facilitating less equivocal comparisons between studies and the pooling of data across studies.

**Electronic supplementary material:**

The online version of this article (doi:10.1186/s12916-015-0470-9) contains supplementary material, which is available to authorized users.

## Background

Healthy ageing and wellbeing are common goals in modern societies. The major demographic shift towards higher proportions of older people within the population in many countries worldwide, and the recognition that much of the costs of health and social care in economically-developed countries is concentrated in the last decade or two of life, have sharpened the research focus on ageing [1].

Research on healthy ageing encompasses: the biological processes contributing to ageing *per se*; the socio-economic and environmental exposures across life which modulate ageing and the risk of age-related frailty, disability and disease; and the development of interventions which may modulate the ageing trajectory [2, 3]. Such research needs measures of biological ageing at the individual level which, in addition to chronological age, can characterise and quantify important functions which are subject to decline at faster, or slower, rates during individual human ageing. Biomarkers of healthy ageing would have utility as surrogate endpoints [4] or outcome measures in trials of interventions designed to extend healthspan and public health-related population surveys would benefit from reliable, readily-measured indices of healthy ageing. However, there is no criterion reference for assessing healthy ageing and this creates difficulties when conducting and comparing research on ageing across studies.

Over the last 50 years [5–7] there have been several attempts to develop markers of ageing but the complexity of the ageing phenotype [8] brings both conceptual and practical difficulties. Despite earlier efforts [9–12], there is currently no universally accepted definition of biomarkers of ageing or criteria for their selection, which has resulted in a lack of robust, validated tools for assessing healthy ageing [6–8]. The American Federation for Aging Research (AFAR) proposed that biomarkers of ageing: ‘1) must predict the rate of aging (it should tell exactly where a person is in their total lifespan and it must be a better predictor of lifespan than chronological age); 2) it must monitor a basic process that underlies the aging process, not the effects of disease; 3) it must be able to be tested repeatedly without harming the person (for example a blood test or an imaging technique); 4) it must be something that works in humans and in laboratory animals, such as mice (so that it can be tested in laboratory animals before being validated in humans)’. Biomarkers fulfilling all of the above AFAR criteria are unlikely to exist [6], and several candidate biomarkers of ageing have emerged in the past few decades but none has proved universally suitable for, or robust in, measuring or predicting the degree of ageing at either population or individual levels [13].

Ageing affects all cells, organs and tissues and, in the majority of body systems, is characterised by the gradual loss of function. When extensive, such functional losses have profound effects which impact on the individual and on family members and carers and have wide-ranging consequences for society. Here we aim to identify a panel of objective biomarkers of healthy ageing in humans where healthy ageing is defined as the maintenance of function for the maximal period of time [3]. Having functionality and pragmatism as our guiding principles, this work focused on those biomarkers which characterise and quantify important functions subject to deterioration in mean levels during ageing and for which there are robust, readily applied tools/instruments for their assessment. We focused attention on the domains of physical capability, cognition, physiological and musculoskeletal functions, and endocrine, immune and sensory functions. However, we recognise that there are important subjective features of the healthy ageing phenotype, including psychological and social wellbeing, which are not covered here [14–16]. In addition, there may be important bidirectional relationships between healthy ageing and wellbeing which are outside the scope of the present work. Our proposed panel of markers was selected from those which are best established, for which there is robust evidence supporting strong associations with ageing phenotypes, and which are likely to be cost-effective and practical for use in larger-scale studies. Most literature focuses on morbidity and mortality as ageing phenotypes or endpoints and there is no independent, criterion reference measure of healthy ageing against which existing or novel biomarkers may be assessed. On this basis and in line with current efforts to standardise definitions and roles of biomarkers [4], the proposed panel of biomarkers comprises a set of surrogate endpoints of important functions influenced by the ageing process.

## Discussion

We aimed to identify objectively assessed biomarkers that are commonly used in population-based studies and applicable in a range of settings (that is, not limited to use in a laboratory/clinic setting), capable of distinguishing between healthy and unhealthy ageing between individuals at older ages, and which change within individuals over time. Where possible, we sought evidence of replication of the proposed marker in different cohorts and using different study designs. The research base in some domains, for example measures of age-related immune function proved to be less well developed than in others, for example measures of physical capability, so that our recommendations in the former domains are more tentative. To help fill the remaining gaps, we also aimed to identify priorities for further research on biomarkers of healthy ageing and these are summarised below in the sections headed ‘Areas lacking adequate evidence’.

**Fig. 1:**
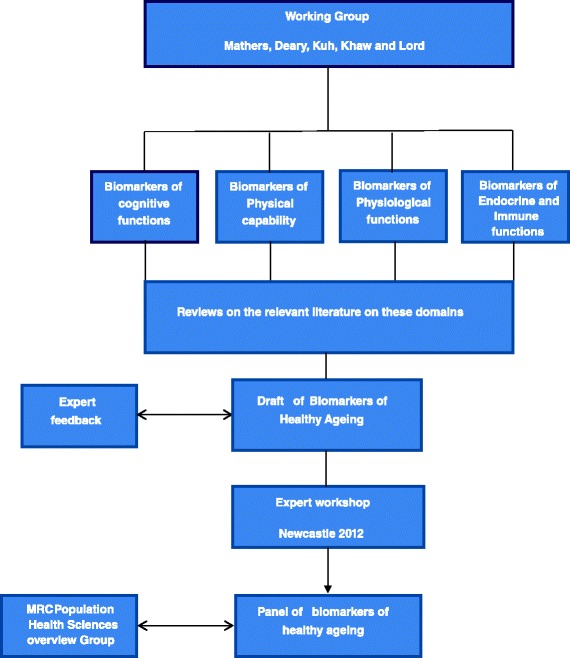
Development and consultation process

The process used to develop recommendations included: 1) undertaking comprehensive reviews of the literature relevant to each domain using, where available, existing systematic reviews, meta-analyses and other authoritative reports such as the recently launched NIH Toolbox for assessment of neurological and behavioural function, which includes test batteries for cognitive and motor function (the latter described here as physical capability) [14]; 2) we invited international experts to comment on our draft recommendations; and 3) we hosted an experts workshop in Newcastle, UK, on 22–23 October 2012, aiming to help capture the state-of-the-art in this complex area and to provide an opportunity for the wider ageing research community to critique the proposed panel of biomarkers (Fig. 1). In this report we also highlight areas needing further research.

This work has been developed at the request of the Medical Research Council (MRC) to address this gap and a version of the report, including details of the evidence used in their derivation, can be found on the MRC website as a resource for the community [17].

### Biomarkers of physical capability

**Fig. 2:**
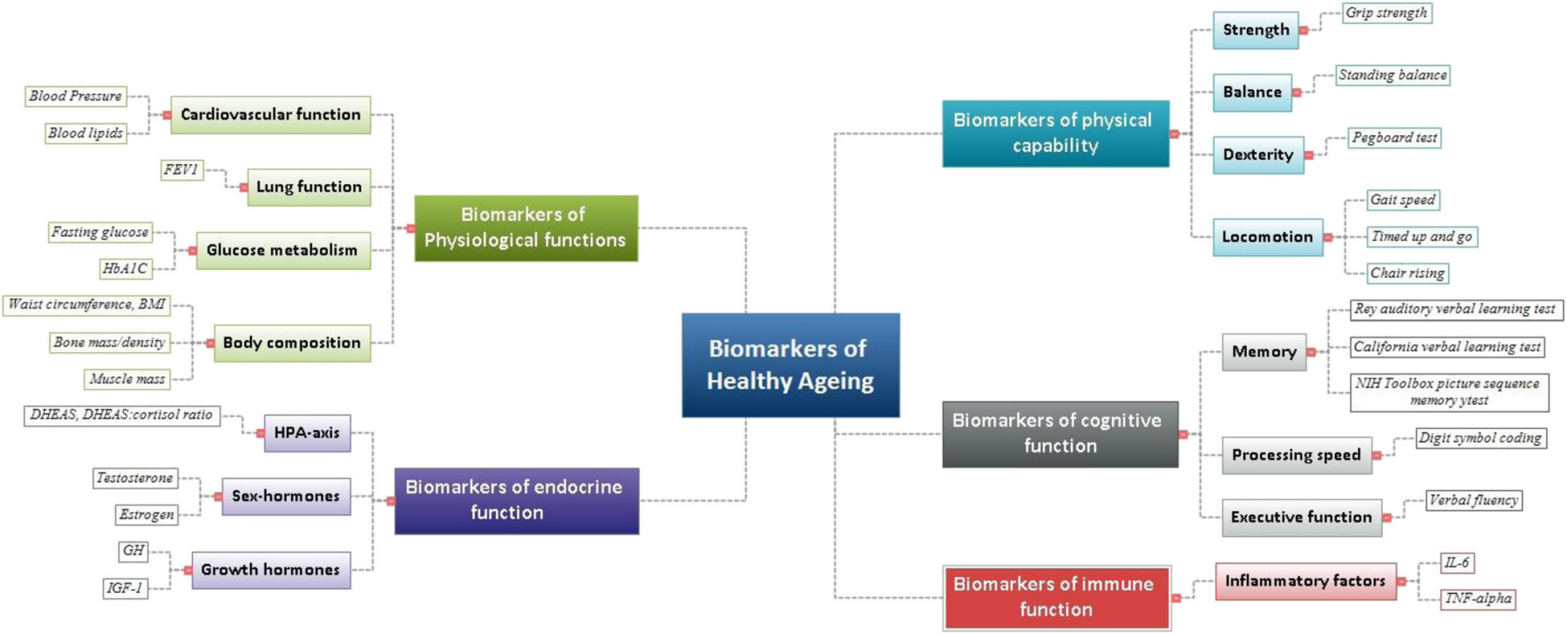
Proposed panel of biomarkers of healthy ageing

Measures of physical capability, that is, a person’s ability to perform the physical tasks of everyday living, are useful markers of current and future health [18]. Guided by previous work by the Healthy Ageing across the Life Course (HALCyon) research collaboration [3] and the NIH Toolbox, we selected four subdomains: locomotor function; strength; balance; and dexterity (Fig. 2 and Additional file 1: Table S1). Physical capability declines progressively in later life with men performing better than women at all ages [19]. Poor performance in tests of grip strength, walking speed, chair rise time and standing balance are associated with higher mortality rates [18, 20]. In addition, lower levels of physical capability are associated with higher risk of cardiovascular disease (CVD), dementia, institutionalisation and difficulties performing activities of daily living (ADLs) [21].

#### Areas lacking adequate evidence

Recent work suggests that there is added value, for the prediction of mortality, in assessing different measures of physical capability in midlife [20]. However, there is currently insufficient evidence to recommend an order of priority for these measures or to define, with confidence, the minimum number of measures that should be made across the range of older ages and for different research questions. More research is also needed on the utility of some measures such as performance in the pegboard test (dexterity) which has been understudied.

There is a need for more studies with longitudinal data on change in physical capability, and need to assess physical capability in relation to other positive aspects of health, such as quality of life, that may be important criteria for healthy ageing [22].

### Biomarkers of physiological function

Complex molecular changes affecting the structure and function of most cells, tissues and organ systems are a hallmark of ageing [8], and changes in their function can be detected by the third or fourth decades of life [23]. Here we focused on biomarkers of lung function, body composition (including bone mass and skeletal muscle), cardiovascular (CV) function and glucose metabolism (Fig. 2 and Additional file 1: Table S2). From age 25 years, forced expiratory volume (FEV1) declines at approximately 32 ml/year in men and 25 ml/year in women, and there are inverse associations between FEV1 and mortality, cognitive function and fractures [24]. Bone mass declines with age, and bone mass or density predicts risk for future fracture and mortality. Large waist circumference, greater body mass index (BMI) and weight-gain in middle age are all associated with higher mortality or lower healthy survival [25, 26]. In addition, low skeletal muscle mass is associated with increased likelihood of functional impairment and disability. Blood pressure (BP) [27, 28] and blood lipids [29, 30] are currently the strongest predictors of CV morbidity and mortality. Increases in diastolic BP and systolic BP are associated with increased risk of CV mortality [27] and high BP in midlife with cognitive decline in later life. Ageing is associated with reduced metabolic capacity exemplified by diminished glucose homeostasis. Raised fasting blood glucose and glycated haemoglobin (HbA1C) are associated with age, CV events and mortality, cognitive impairment, and dementia, in non-diabetics.

#### Areas lacking adequate evidence

Emerging biomarkers, for example fibrinogen, plasma cystatin C and brain natriuretic peptide, have been associated with increased risk of CV events and mortality, but it is uncertain if these offer advantages over well-established biomarkers. More research is needed on whether monitoring biomarkers over longer time periods, for example glucose concentration and ambulatory BP over 24 hours, or in response to a challenge, improves their predictive value.

### Biomarkers of cognitive function

Cognitive decline may limit independence and signal dementia [31], and, although debated [32], evidence indicates that the onset of cognitive decline is detectable relatively early in adulthood, for example from around 45 years of age or earlier in some functions [33]. We focused on cognitive domains assessed widely in human ageing studies and employed in the NIH Toolbox. We identified nine domains together with tests commonly used for their assessment (Additional file 1: Table S3). Based on current evidence, three domains – executive function, processing speed and episodic memory – are a possible minimum set of domains to be assessed in ageing studies (Fig. 2). If assessment time allows, tests of crystallised cognitive ability and non-verbal reasoning would be useful additions. Executive function is markedly affected by ageing [34], exhibiting an inverted U-shape pattern across the lifespan. Processing speed declines progressively with age [35] and is associated with greater mortality risk [36], CVD and respiratory disease [37]. In addition, episodic memory is sensitive to brain ageing and declines in individuals with mild cognitive impairment and neurodegenerative diseases [38]. A standard deviation advantage in memory is associated with 21 % reduction in mortality risk among older individuals [39].

#### Areas lacking adequate evidence

To date, computer-based tests are not widely used in major cohorts; availability of tools such as the NIH Toolbox and the imperative to increase cost-effectiveness are likely to drive the migration to digital methodologies. This will require that tests are supported by on-going technical development to ‘future-proof’ operating systems and hardware. Where tests are administered repeatedly in the same individuals problems associated with practice and familiarity need to be addressed. The issue of co-variance among cognitive tests needs more attention because those who score well on one test tend to score well on others [40]. Salthouse and others have highlighted that the causes of cognitive ageing might affect the variance shared by tests or domains or the variance in a specific test or domain [40].

### Biomarkers of endocrine function

Age-related changes in the endocrine system, particularly the sex hormones, are well recognised and have established causal links with health outcomes. We focused on sex hormones, the HPA axis, growth hormone IGF-1, melatonin, adipokines and thyroid hormones (Fig. 2 and Additional file 1: Table S4). Strong consensual evidence from longitudinal studies indicates that testosterone, estrogen, DHEAS and growth hormone IGF-1 are linked with risk of premature mortality and physical frailty [41]. For some biomarkers, the relationship with ageing appears to be non-linear, for example both high and low IGF-1 are related to greater mortality rates. DHEAS declines with age from the third decade onwards and low DHEAS is associated with increased mortality in older subjects with concurrent frailty. Hormone replacement studies suggest causal links for both testosterone and estrogen and risk of physical frailty and bone health [42, 43]. Cortisol is associated with age-related disease and disability [44], and abnormal cortisol secretion patterns are associated with increased BP, impaired glucose metabolism and increased incidence of CVD and type 2 diabetes in men [45].

#### Areas lacking adequate evidence

Longitudinal evidence is needed to enhance understanding of the relationships between cortisol, DHEAS, cortisol:DHEAS ratio, adipokines (adiponectin, leptin, ghrelin), somatostatin, and ageing, frailty and mortality.

### Biomarkers of immune function

Whilst the field of immunology is well developed, the study of age-related decline in immunity, termed immunosenescence, is more recent [46]. Here we focused on age-related immune function and inflammatory factors (Fig. 2 and Additional file 1: Table S4). Longitudinal studies comparing immune cells or function with mortality, or with age-related functions such as infection rates or vaccination responses, are scarce [47]. Two octogenarian and nonagenarian studies assessing immune markers (T-cell phenotype, cytomegalovirus serostatus and pro-inflammatory cytokine status) with subsequent mortality have been the basis for the development of the immune risk profile (IRP) [48], which is associated with mortality in those over 60 years [49]. A limitation of the IRP is its narrow scope since it does not consider innate immune factors such as natural killer cell (NK cell) function, which is linked with infection rates and mortality. The best studied aspect of immunosenescence is the age-related increase in systemic inflammatory cytokines, inflammageing [50]. Higher plasma concentrations of IL-6 and TNF-α are associated with lower grip strength and gait speed in older adults [51]. Centenarians show fewer signs of ageing of the immune system although some inflammageing is seen.

#### Areas lacking adequate evidence

Longitudinal studies should examine relationships between number and function of T cells, neutrophils, NK cells, B cells, and mortality, risk of age-related disease and wellbeing in later life. Given the switch from lymphoid to myeloid cell production with age, the lymphocyte/granulocyte ratio is a potentially useful biomarker of healthy ageing. The IRP needs validation in younger people and should be expanded to include measures of immune function such as infection incidence or vaccination response. Telomere length in leukocytes, including lymphocytes and monocytes, has received much attention. Despite its association with ageing in several cohorts, it is likely that shortened telomeres are also a marker of infection frequency so that leukocyte telomere length may not be a reliable index of biological ageing. Further studies of telomere length and ageing should include investigation of exposure to infections and CMV seropositivity as possible confounders. In the Newcastle 85+ Study, telomere length was uninformative about health status [13].

### Sensory functions as potential biomarkers of ageing

Sensory functions are critical for normal levels of independence, for interactions with others and to facilitate enjoyment of life’s experiences. Loss of these functions is more prevalent in older adults, with loss of audition and vision being the most prominent. The prevalence of visual impairment increases with age and may reduce the ability to undertake daily activities such as reading, and may limit mobility and social interactions. Olfactory acuity declines with age, is more common among men, and has been proposed as an indicator of brain integrity in older people. Smell dysfunction is among the earliest ‘preclinical’ sign of neurodegenerative diseases such as Alzheimer’s disease and sporadic Parkinson’s disease [52], and is associated with mortality in the National Social Life, Health and Aging Project [53]. The NIH Toolbox [14] measures audition, vision, olfaction, gustation, vestibular function and pain. Most of these functions, with the exception of pain, decrease across the lifespan, and sensory changes may overlap with changes in cognitive and motor functions. However, the predictive value of measures of sensory function for age-related health outcomes remains uncertain as does the opportunity to modulate ageing-related changes in sensory function through lifestyle or other interventions. Further evidence will be needed before sensory measures can be recommended with confidence as reliable markers of healthy ageing.

## Summary

We have proposed a panel of measures of healthy ageing which we hope will be of utility to researchers undertaking cross-sectional and longitudinal studies, and, potentially, as surrogate endpoints or outcome measures for interventions to enhance healthy ageing. We have selected these biomarkers with the concept that, for pragmatic purposes, healthy ageing can be operationalised as preserved physical, cognitive, physiological, endocrine, immune and metabolic functions. The proposed panel of biomarkers of healthy ageing are well-established individually, are commonly used in several settings and study designs, have analytic and clinical validity and relevance, and some have proven value in clinical practice and health-related research. In addition, for some, their predictive value has been replicated in different cohorts, and therefore are currently the strongest surrogate endpoints of important ageing-related functions and the ageing process itself [4]. The proposed panel includes biomarkers such as blood pressure, fasting glucose and HbA1C, bone mineral density, and blood lipids, each of which is considered disease-defining and do not match item two of the AFAR definition criteria. However, these biomarkers appear to be predictive of biological age and of the rate of ageing in younger healthy subjects [54]. In these examples, changes in the biomarkers appear to reflect subtle changes in ageing-related processes (likely driven by differences in the rate of accumulation of molecular damage) rather than frank disease. We are aware that there is scientific interest in a number of ‘emerging’ biomarkers of ageing, some of which are being explored in research initiatives such as the Europe-wide MARK-AGE consortium [55]. As evidence of their utility becomes available, further biomarkers could be added to, or substituted for items in, the current panel.

From the available evidence it was not possible to rank the domains or sub-domains proposed nor to suggest how information from the various domains might be aggregated to provide a ‘healthy ageing’ score – even assuming that such a score is conceptually valid or of practical utility. However, combinations of some of these biomarkers appear to predict biological age and the rate of ageing among young adults [54, 56], as well as frailty [57, 58], and further research in this area should help to identify whether the proposed biomarkers can be combined to produce an overall ‘ageing score’ and the circumstances in which such a score has practical utility.

A further generic limitation of our work is uncertainty about the validity in very old people of putative biomarkers of healthy ageing which appear robust in younger-old individuals. Indeed, in some cases the reverse may apply, for example higher BP in very old people may be protective [13]. Here we have used a restricted canvas to focus on biologically well-understood objective measures, which could be employed globally in a wide range of different types of study. Adoption of this approach may facilitate the comparability, and pooling, of data from a greater number of studies than is possible at present and so enhance research on healthy ageing.

## Additional file

Additional file 1: Table S1. Summary of recommended biomarkers in the physical capability domain. **Table S2.** Summary of recommended biomarkers in the physiological domain. **Table S3.** Summary of biomarkers in the cognitive domain relevant to ageing. **Table S4.** Summary of recommended biomarkers in the endocrine function domain. **References**. (DOCX 24 kb)

## Abbreviations

ADLs: Activities of daily living
AFAR: American Federation for Aging Research
BMI: Body mass index
CV: Cardiovascular
CVD: Cardiovascular disease
DHEAS: Dehydroepiandrosterone sulphate
FEV: Forced expiratory volume
HALCyon: Healthy Ageing across the Life Course
HbA1C: Glycated haemoglobin
IL: Interleukin
IRP: Immune risk profile
MRC: Medical Research Council
NK cell: Natural killer cell
TNF: Tumor necrosis factor
