# Supplementary material for: A proposed panel of biomarkers of healthy ageing
Source: BMC Med. 2015 Sep 15;13:222. doi: 10.1186/s12916-015-0470-9 (PMC4572626; doi:10.1186/s12916-015-0470-9)
Supplement: Additional file 1: Table S1. — Summary of recommended biomarkers in the physical capability domain. Table S2. Summary of recommended biomarkers in the physiological domain. Table S3. Summary of biomarkers in the cognitive domain relevant to ageing. Table S4. Summary of recommended biomarkers in the endocrine function domain. References. (DOCX 24 kb) [file 12916_2015_470_MOESM1_ESM.docx]

**Additional file 1**

| **Table S1.** **Summary of recommended biomarkers in the physical capability domain** | | | | |
| --- | --- | --- | --- | --- |
| **Domain** | **Tool/**  **measure** | **Feasibility of use** | **Prediction of outcome** | **Approximate costs/equipment*** |
| **Locomotor function** | Gait (Walking) speed | +++ | Mortality  Falls  +++ | Stopwatch (£6)  Measuring tape (£6)  Marker tape (£5)  Plastic cones (£5)  Clipboard (£3) |
|  | Timed get up and go | +++ | Mortality  +++ |  |
|  | Chair rising | +++ | Mortality  +++ |  |
| **Strength** | Grip strength | +++ | Mortality  +++ | Jamar Hand Dynamometer  Hydraulic from £215 +VAT  Digital from £265 +VAT |
| **Balance** | Standing balance (One leg stand, tandem stands) | +++ | Mortality  +++ | ***AIREX Balance Pad Elite £90*  ***Accelerometer £approx £100* |
| **Dexterity** | Pegboard test | +++ | Evidence lacking | Rolyan®9-Hole Peg Test Kit (board+pegs+stopwatch) £72  (replacement pegs £15) |

Degree of feasibility of use and prediction of outcomes: +++ strong; ++ moderate, + low

*Costs were obtained from a number of online sellers in December 2014.

***Note. Although accelerometers are not yet commonly used to asesss balance in large epidemiological studies, the NIH toolbox balance test requires an AIREX Balance Pad and an accelerometer.*

| **Table S2** **Summary of recommended biomarkers in the physiological domain** | | | |
| --- | --- | --- | --- |
| **Subdomain** | **Tool/test** | **Feasibility of use** | **Prediction of outcome** |
| **Lung function** | Spirometry:  Forced Expiratory Volume in 1 sec(FEV1) | +++ | Mortality, cardiovascular events, fractures, functional health, cognition |
| **Bone health** | Bone density, bone mass hip: Dual X ray Absorptiometry | ++ | Mortality, fractures, CVD |
|  | Ultrasound: broadband ultrasound attenuation (BUA) at heel | +++ |  |
| **Skeletal Muscle** | Estimated leg muscle mass Dual X ray Absorptiometry | ++ | Uncertain |
|  | Estimated muscle mass Body impedance | +++ | Mortality |
|  | Abdominal fat Waist circumference | +++ | Mortality, cardiovascular events, |
|  | Body mass Body Mass Index  Body weight | +++ | Mortality, cardiovascular events, |
| **Cardiovascular function** | Systolic blood pressure  Sphygmomanometry | +++ | CVD, mortality |
|  | Lipid profile: total cholesterol, LDL-C, HDL-C, Triglycerides  Biochemistry assay | ++ | CHD |
| **Glucose metabolism** | Glycated haemoglobin  Fasting plasma glucose  Biochemistry assay | ++ | Mortality, CVD |

Degree of feasibility of use: +++ strong; ++ moderate, + low

| **Table S3. Summary of biomarkers in the cognitive domain relevant to ageing** | | | | | | |
| --- | --- | --- | --- | --- | --- | --- |
| **Subdomain** | **Tool/Test** | **No. studies using the test** | **Feasibility of use** | **Time to administer*** | **Age range (norms)*** | **Cost*** |
| Executive Function | Verbal Fluency^a^ | 41 | +++ | ~5 mins | 6-95 | Free |
| Processing Speed | Digit-Symbol Coding^b^ | 18 | +++ | ~5 mins | 16-89 | WAIS III = $530.25 (£328), WAIS IV = $1,145 (£709) |
| Working Memory | Digit Span backward | 15 | +++ | ~5 mins | 16-89 | WAIS III = $530.25 (£328), WAIS IV = $1,145 (£709) |
| Crystallised Ability | Boston Naming Test | 12 | +++ | ~10-20 mins | 20-85 | $121 (£75) |
| Attention | Stroop^c^ | 11 | +++ | ~5 mins | 8-89 | Free - $150 (£93) |
| Visuo-Spatial Ability | Block Design | 10 | ++ | ~10-15 mins | 16-89 | WAIS III = $530.25 (£328), WAIS IV = $1,145 (£709) |
| Reasoning | Raven’s Progressive Matrices | 9 | +++ | ~40-60 mins | 6.5-70+ | SPM = $206 (£128), APM = $243 (£151), Comprehensive kit = $874 (£541) |
| Verbal Memory & Learning | Rey Auditory Verbal Learning Test^c^ | 7 | +++ | ~10-15mins | 6-89 | Free (English version) |
| Visual Memory | Benton Visual Retention Test | 6 | +++ | ~5-20 mins | 8-80+ | 5th Ed = $242 (£148) |

Degree of feasibility of use: +++ strong; ++ moderate, + low

*= Information from several sources including Strauss *et al*. (2006)^[1](#_ENREF_1)^, Lezak *et al*. (2012)^[2](#_ENREF_2)^ Wechsler (1997)^[3](#_ENREF_3)^, Wechsler (1997) and [www.pearsonassessments.com](http://www.pearsonassessments.com).

*Key: SPM = Standard Progressive Matrices, APM = Advanced Progressive Matrices, WAIS = Wechsler Adult Intelligence Scale, WMS = Wechsler Memory Scale; we give prices for the whole battery, but note that this covers many tests in addition to those single tests identified in the table.*

^a^This is often letter fluency or semantic fluency; ^b^The reverse—Symbol-Digit Modalities is also used and there are some free versions of this type of test; ^c^There are many versions of this type of test;

| **Table S4** **Summary of recommended biomarkers in the endocrine function domain** | | | | |
| --- | --- | --- | --- | --- |
| **Domain** | **Tool/Test** | **Relationship to aging** | **Predictor**  **of mortality** | **Association with frailty** |
| **Endocrine function** | Adiponectin | +++ / | +++++ | + |
|  | DHEAS:Cortisol ratio | ++ | + | ++ |
|  | DHEAS | +++++ | + | ++ |
|  | Growth Hormone/IGF-1 | +++++ | ++ | ++ |
|  | Leptin | ++ | + | + |
|  | Ghrelin | + | + | + |
|  | Melatonin | ++++ | + | + |
|  | Estrogen/ Oestrogen | +++++ | + | +++++ |
|  | Somatostatin | + | + | + |
|  | Testosterone | +++++ | + | ++++ |
|  | Thyroid hormones | ++ | + | +++ |
|  |  |  |  |  |
| **Immune function** | B Cells | +++++ | + | + |
|  | CMV sero+ve | ++++ | ++ | + |
|  | C-reactive protein | +++++ | +++++ | + |
|  | Dendritic cells | + | + | + |
|  | IL-6 | +++++ | +++++ | +++ |
|  | Natural Killer Cells | +++++ | + | ++ |
|  | Neutrophils | +++++ | + | + |
|  | Telomere length | +++++ | +++++* | + |
|  | T Cell phenotype | +++++ | + | + |

Supporting evidence: +++++ very strong; ++++ strong, +++ moderate, ++ low, + very low or none; NA not applicable

* large amount of evidence, but mixed results.

**References**

1. Strauss E, Sherman EMS, Spreen O, Spreen OCont. *A compendium of neuropsychological tests : administration, norms, and commentary*. 3rd ed. ed. New York ; Oxford: Oxford University Press, 2006.

2. Lezak MD, Howieson DB, Loring DW. *Neuropsychological assessment*. 4th ed. New York: Oxford University Press, 2004.

3. Wechsler D. *Wechsler Memory Scale - Third Edition (UK): Administration and scoring manual.* London, 1997.
